# The effect of subcutaneous injection of methylprednisolone acetate and lidocaine for refractory postherpetic neuralgia: a prospective, observational study

**DOI:** 10.1002/hsr2.271

**Published:** 2021-04-08

**Authors:** Duc Thuan Nguyen, Thanh Chung Dang, Quang An Nguyen, Trung Duc Le, Thi Dung Hoang, Thi Ngoc Truong Tran, Ta Hai Ninh Duong, Van Tuan Nguyen, Van Quan Le, Tien Ung Hoang, Minh Tuan Duong, Dinh Son Nhu, Viet Nga Phan

**Affiliations:** ^1^ Department of Neurology Military Hospital 103, Vietnam Military Medical University Hanoi Vietnam; ^2^ Department of Pathophysiology Vietnam Military Medical University Hanoi Vietnam; ^3^ Stroke Center Phu Tho General Hospital Viet Tri Phu Tho Province Vietnam; ^4^ Department of Rehabilitation Military Hospital 103, Vietnam Military Medical University Hanoi Vietnam; ^5^ Department of Functional Exploration Military Hospital 103, Vietnam Military Medical University Hanoi Vietnam; ^6^ Hospital of the 4th Military District Nghe An Vietnam

**Keywords:** refractory postherpetic neuralgia, subcutaneous injection, methylprednisolone acetate, lidocaine

## Abstract

**Background:**

Postherpetic neuralgia (PHN) is the most common and bearable complication of herpes zoster (HZ). This pain may have negative impact on the patient's all aspects of daily life and health‐related quality of life (HRQOL). Despite numerous advances in treatment, many patients remain resistant to the current therapy options. It is the first time subcutaneous injection of methylprednisolone acetate and lidocaine has been used to treat *refractory* PHN. We report the results of this treatment evaluating pain relief and HRQOL improvement in this disorder.

**Methods:**

A total of 43 patients with refractory PHN was enrolled in the observational study. All patients received daily subcutaneous injection of methylprednisolone acetate and lidocaine for 10 consecutive days. The severity of pain was assessed by using Visual Analog Scale (VAS), and 36‐Item Short Form Survey (SF‐36) was applied to evaluate HRQOL. Assessment of the pain and HRQOL was carried out at baseline and posttreatment at 4 weeks as well as 6 and 12 months.

**Results:**

At baseline, all patients experienced severe PHN with average VAS scores of 8.44 ± 0.85 (minimum 7; maximum 10). At 4 weeks, 6 months, and 12 months after treatment, the pain had significantly decreased (*P* < .001), and all subjects showed significant improvement in all eight domains of HRQOL. No major adverse events associated with the subcutaneous injection were observed.

**Conclusions:**

Our results indicate that subcutaneous injection of methylprednisolone acetate and lidocaine can be an effective and safe treatment for PHN.

## 1 INTRODUCTION

Postherpetic neuralgia (PHN), a feared complication of herpes zoster (shingles), is commonly defined when herpes‐associated pain persists 3 months or longer following herpes zoster outbreak.^1^, ^2^ The typical characteristic of this chronic neuropathic pain is various including constant aching or intense burning pain, a paroxysmal, lancinating pain. It is also frequently accompanied by allodynia (pain from usually non‐noxious stimuli) and/or hyperalgesia (increased response caused by stimuli that normally provoke pain).^3^, ^4^ The negative impacts on patients' activities of daily living and health‐related quality of life (HRQOL) may be devastating.^5^ It affects not only physical but also functional and psychological health of the patients and even spouses and relatives.^6^, ^7^, ^8^ Additionally, PHN has a negative effect on healthcare system as well as a whole society.^5^ Despite numerous advanced treatments, PHN can be prolonged for many months or even years and refractory to currently available therapy options.^5^, ^9^ Subcutaneous injection of corticosteroids and local anesthetics has been administered to prevent and treat PHN^10^, ^11^, but not yet to be used to treat refractory PHN. We carried out this study to assess the effectiveness of subcutaneous injection of methylprednisolone acetate and lidocaine on this condition.

## 2 METHODS

### 2.1 Study population

This was a prospective, observational research conducted in Military Hospital 103, Hanoi, Vietnam, with a 1 year follow‐up. During the period from May 2016 to October 2018, a total of 46 patients with refractory PHN were screened at the time of pretreatment examination and 43 patients were enrolled in the entire study (up to 12 months after treatment). All of them gave the written informed consent to participate in the study. The work was approved by Ethics Committee of Military Hospital 103 (code: 140/2016/IRB‐MH103).

We defined refractory PHN as constant aching or intense burning pain, a paroxysmal and lancinating pain with allodynia and/or hyperalgesia that was in the restriction of dermatome involved to original herpes zoster,^12^ and did not respond to conventional treatment including anticonvulsants (gabapentin and pregabalin), tricyclic antidepressants (amitriptyline and nortriptyline), topical agents (5% lidocaine patch), tramadol (in combination with acetaminophen), or patients had contraindications to or intolerance of the drugs. The patients were to be included only if the pain duration was longer than 3 months after the resolution of the HZ‐associated skin rash. We excluded the cases with one or more of the following conditions: coagulation disorders due to any origin; bacterial or fungal infection in the involved dermatome; polyneuropathy; neuropathic pain due to other causes, known allergy to methylprednisolone acetate or lidocaine; serious immunodeficiency diseases (eg, HIV and cancer).

### 2.2 Protocol

All eligible patients received a combination of methylprednisolone acetate (5‐10 mg) and lidocaine 2% (6‐12 mL), depending on the severity and area of pain. The affected site was divided into a chessboard form with 0.3 to 1.0 mL subcutaneously injected at each point to cover the entire pain area (Figure S1).

The injection was once daily administered with a 25 G needle for 10 consecutive days. The injection points can be changed if pain at the previous injected point increases or a large hemorrhage occurred.

The severity of pain was assessed by using VAS. It consists of a straight horizontal line of 100 mm length. The ends are defined as the extreme limits of the pain “no pain at all” on the left end (0 cm) and “as bad as it could be” on the right end of the scale (100 mm). The VAS was frequently used for measurement of pain in recent years.^13^ The severity of pain was categorized as severe (VAS score: 7‐10), moderate (VAS score: 4‐6), and mild (VAS score: 0‐3). Pain relief was classified as excellent (>80% relief), good (50 to 80% relief), fair (20 to 49% relief), or poor (<20% relief). HRQOL was measured by 36‐Item Short Form Survey (SF‐36), which measures eight concepts of health status: physical functioning (PF: 10 items), role emotional problems (REP: 3 items), role physical problems (RPP: 4 items), social functioning (SF: 2 items), bodily pain (BP: 2 items), mental health (MH: 5 items), vitality (VT: 4 items), and general health perceptions (GHP: 5 items). For each domain of health status, scores are summed and transformed to a scale from 0 (worst HRQOL) to 100 (best HRQOL).^14^ The validity of the SF‐36 for use in HZ and PHN patients has been proved.^15^, ^16^

### 2.3 Time of evaluation

At the time of pretreatment (baseline), the demographic data of the study population (age of disease onset, sex, comorbidities, and location of herpes zoster lesion) were collected. In addition, VAS scores and scores for each of the eight items on the SF‐36 scale were also counted. At the time of completion of treatment, only VAS scores were calculated and recorded. At follow‐up 4 weeks, 6 months, and 12 months after treatment: scores of VAS and the eight concepts on the SF‐36 scale were again counted and collected.

#### 2.3.1 Statistics

**FIGURE 1:**
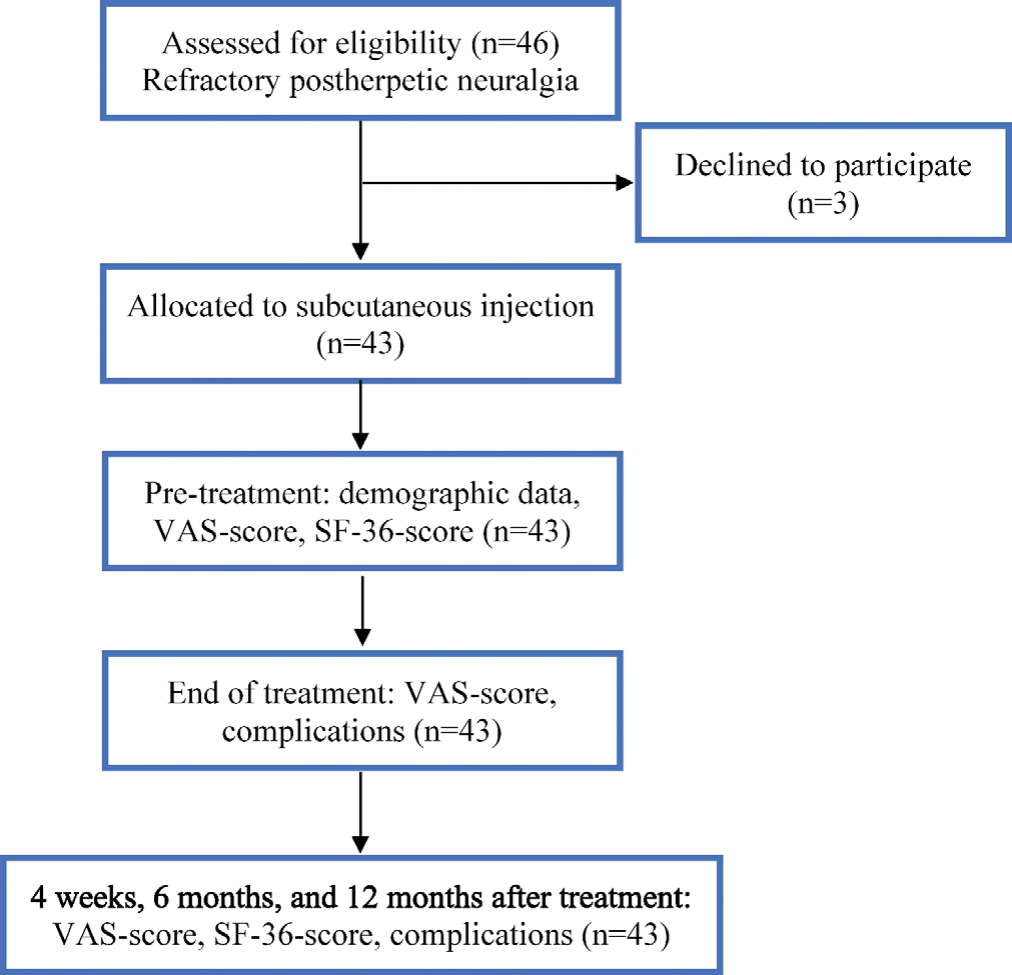
Research profile

The data were entered into Excel spreadsheet and then transferred to SPSS software for analysis and processing. The data are expressed as mean, standard deviation, maximum value, minimum value, number, and percentage. Numeric data were expressed as mean ± SD, and non‐numeric data were presented as percentage. The difference between the averages is assessed by the Wilcoxon signed rank sum test. The continuous line graphs were used to present the change of pain severity (VAS score) and improvement of HRQOL (SF‐36 score) over time. *P* < .05 was considered statistically significant. The data were analyzed on SPSS software (version 22.0) for Windows operating system (SPSS Inc. IBM Company) (Figure 1).

## 3 RESULTS

During the 18‐month enrollment period, a total of 46 PHN patients were screened and 43 patients recruited in the study. Three of them were excluded for declining to participate in the study, and 43 subjects completed the 12‐month follow‐up. The clinical and demographic characteristics of the subjects before the start of the research are presented in Table 1.

**TABLE 1:** Demographic features of the study population

Feature		
Age (year: mean, SD)		64.2 ± 8.8
Sex: *n* (%)	Male	17 (39.5)
	Female	26 (60.5)
Duration of pain after eruption healing (month: mean, SD)		5.9 ± 2.4
Comorbidities: *n* (%)	Hypertension	12 (27.9)
	Diabetes mellitus	2 (4.7)
Location of herpes zoster lesion: *n* (%)	Supraorbital	4 (9.3)
	Cervical	5 (11.6)
	Upper thoracic (D1‐D8)	21 (48.8)
	Lower thoracic (D9‐D12)	7 (16.3)
	Femoral	2 (4.7)
	Lumbar	1 (2.3)
	Forearm	2 (4.7)
	More than one location of lesion	1 (2.3)

**FIGURE 2:**
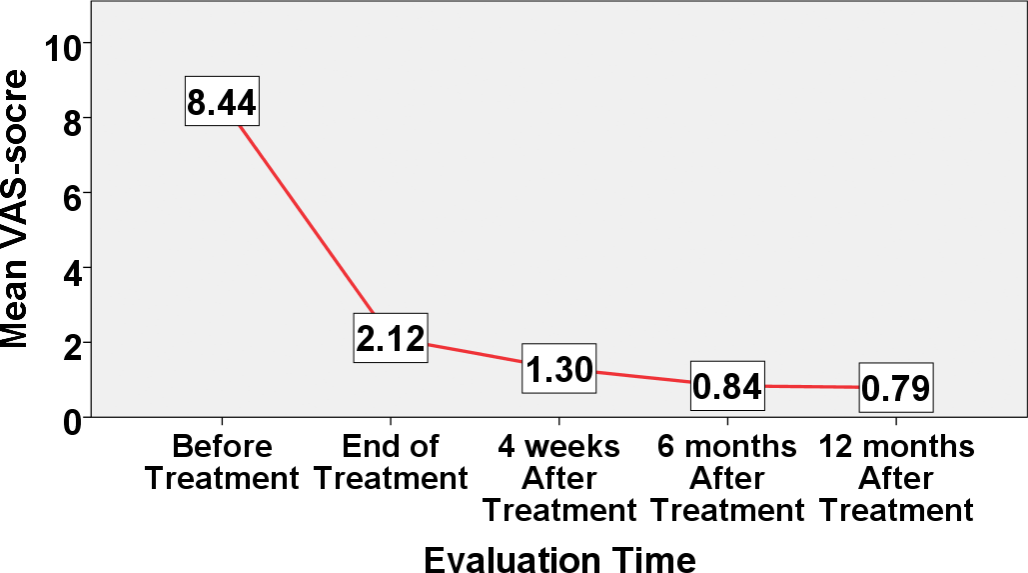
Pain severity of study population before and after treatment

Figure 2 shows the intensity of pain at different time points. The VAS scores were significantly lower in all subjects at all four follow‐up time points (*P* < .001). Further analysis of the data, all patients had an excellent pain relief during the 12‐month follow‐up. At the end of the study, 17/43 (39.5%) individuals had no pain anymore, and none of them reported recurrent pain at all posttreatment assessment.

Table 2 presents the pain grade at different time points. Compared to before treatment, at the end of treatment, the patient's pain grade changed significantly (mild pain was seen in 97.7% of patients) (*P* < .001). Four weeks after treatment, all patients had only mild pain and this outcome was maintained across a period of 12 months.

**TABLE 2:** Change of pain grade of study population before and after treatment

Pain grade	Before treatment	End of Treatment	4 weeks after treatment	6 months after treatment	12 months after treatment	*P* ^a^
Severe (VAS:7‐10)	97.7%	0%	0%	0%	0%	<.001
Moderate (VAS:4‐6)	2.3%	2.3%	0%	0%	0%	<.001
Mild (VAS:0‐3)	0%	97.7%	100%	100%	100%	<.001

^a^ Wilcoxon signed‐rank test.

**FIGURE 3:**
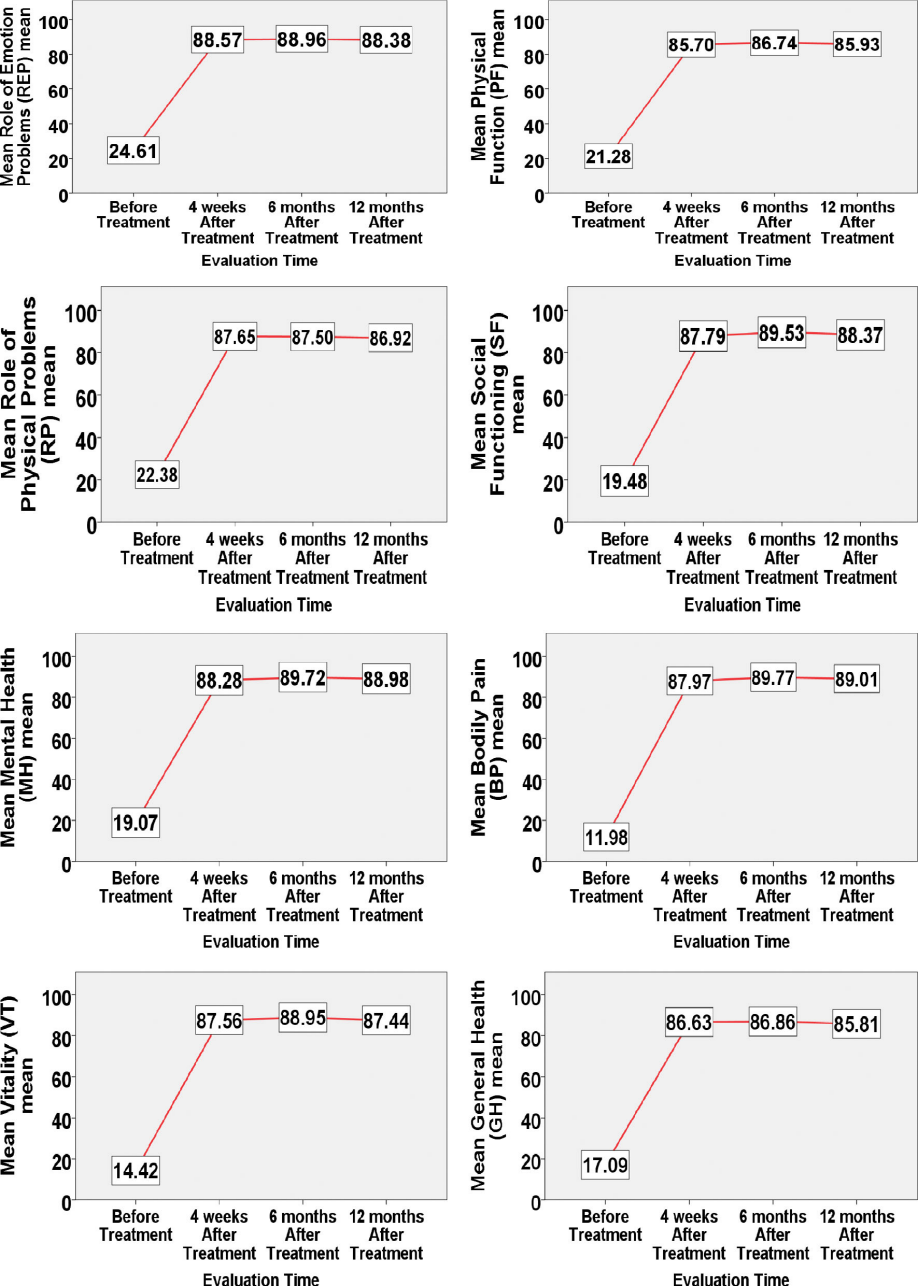
Health‐related quality of life before treatment and 12‐month follow‐up

The HRQOL, as measured by SF‐36, showed statistically significant improvement in all eight domains from baseline at any follow‐up evaluation (Figure 3 and Table S1).

### 3.1 Adverse effect

Local adverse effect reactions such as small subcutaneous bleeding (Figure S2) and pain at the injection points were noted in 27/43 (62.8%) and 31/43 (72.1%) of patients, respectively. Systematic complications, including vertigo and weight gain, due to water retention were observed in 4/43 (9.3%) patients. Abscesses, cutaneous atrophy, scarring, lidocaine‐related acute intoxication, arrhythmia, and dysesthesias were not reported.

## 4 DISCUSSION AND CONCLUSION

PHN is the most common and troublesome chronic complication associated with HZ.^17^, ^18^ Managing PHN, so far, can be still a challenging process due to the lack of the therapeutic approach with both efficacy and tolerability.^3^ Current guidelines usually recommended conventional drugs for pain relief, including gabapentinoids (pregabalin and gabapentin), tricyclic antidepressants (amitriptyline, nortriptyline, desipramine, and maprotiline), tramadol, or opioids as first‐ or second‐line treatment of PHN.^19^, ^20^ However, these systemic agents cannot be widely used as a long‐term regimen because of their side effects, especially in elderly patients who have the age‐related (associated) physiologic changes, comorbidities, and polypharmacy (among whom the PHN is most prevalent).^21^ Topical medications (5% lidocaine patch and capsaicin) are also approved for the treatment of PHN in European countries and the United States. However, evidence in support of their efficacy is often lacking. In addition, both drugs using alone are only reasonable to consider as first‐line treatment for mild pain.^18^ Therefore, patients with severe pain like those in our study cannot be beneficial from this therapy. With regard to the interventional procedures for the treatment of PHN patients, the results of a systematic review indicated that most of them were recommended as grade B because of the absence of a high level of evidence except for intrathecal methylprednisolone injection, but this intervention should be only conducted with great caution and careful comprehensive assessment.^4^

Currently, in Vietnam, conventional regimens including anticonvulsants (gabapentin and pregabalin), tricyclic antidepressants (amitriptyline and nortriptyline), topical agents (5% lidocaine patch), and tramadol (in combination with acetaminophen) are available and acceptable for treatment patients with PHN. All patients who prior to being recruited into our research had been tried for treatment with the abovementioned drugs in other hospitals, but they showed a treatment failure. Their pain was still not well controlled and remained severe. In our study, the mean score of pain severity according to VAS before intervention was 8.44 ± 0.85 (minimal VAS score of 7; maximal VAS score of 10). Although opioids and intrathecal methylprednisolone are recommended according to American and European guideline (Level A, Class I and II) for therapy of this condition,^19^, ^20^ they have still not been established in our country.

In the present study, the administration of subcutaneous injection of methylprednisolone in combination with lidocaine for 10 consecutive days had shown an excellent effect on pain reduction (measured by a VAS) and improvement of the quality of life (on a SF‐36 scale) of 43 patients with *refractory* PHN.

In terms of pain‐relieving effect, we found that within initial 2 to 3 days of the intervention, pain‐relieving efficacy could not be observed, even increasing pain occurred at the injection points. Analgesic improvement with the regimen started 4 to 5 days during intervention, desired results were reached at the end of the procedure (on the 10th day of treatment course), and analgesic effect lasted throughout the 12 months of follow‐up. At the end of the study, although 26/43 (61.5%) of patients remained mild pain and pain completely disappeared in only 17/43 (38.5%) of individuals, all of them indicated satisfaction with the efficacy of this pain treatment. Only one 74‐year‐old female patient with HZ at lower thoracic (D9‐D12) area, 12 months after intervention, reported intermittent mild intra‐abdominal pain with VAS‐score of 3 (before treatment VAS‐score of 9), but the cutaneous symptoms cleared completely with this treatment. Therefore, the patient did not require any further intervention.

It has been known that neural and perineural inflammation, peripheral sensitization of primary afferent sensory nerve fibers, and central sensitization are thought to be responsible for PHN.^22^, ^23^, ^24^ Lidocaine is proposed to block voltage‐gated sodium channels of dysfunctional small myelinated Aδ‐ and unmyelinated C‐fibers resulting in reduction in ectopic impulses and decreased spontaneous pain, allodynia, and hyperalgesia.^25^, ^26^ Corticosteroids act as an anti‐inflammatory agent, which has been suggested to minimize nerve damage and stabilize neural‐cell membranes and suppress ectopic neural discharges of C‐fibers, and thereby relieve HZ‐associated pain.^27^, ^28^ Additionally, among available corticosteroids, methylprednisolone acetate has been found to be the least neurotoxic agent in humans.^29^ Therefore, this drug was used in our study. An intervention course of 10 consecutive days was applied because a pain‐relieving permanent effect can be obtained with a series of 8 to 12 infiltrations on consecutive days.^28^ Moreover, little additional benefit can be expected after the initial 12 to 14 intervention days,^30^ and longer intervention duration, based on our experiences, frequently leads to systematic side effects of corticosteroid (eg, weight gain due to fluid retention).

The mixture of corticosteroids and local anesthetics has been broadly used to treat numerous disorders with neuropathic pain, including treatment and prevention of HZ and PHN,^4^, ^10^, ^11^, ^28^, ^29^, ^30^, ^31^ but to our best knowledge, this is the first time a combination of injectable methylprednisolone and lidocaine have been administered for treating *refractory* PHN. In the literature, we found a small number of studies with other options to manage this condition. In a work conducted by Kotani and colleagues in 2000, that using the intrathecal methylprednisolone for therapy, PHN‐patient who was resistant to conventional regimens and showed a good and excellent result.^32^ However, this treatment method presented opposite results in another later investigation.^33^ In another publication in 2014, the authors presented a 64‐year‐old male patient with an intractable lower thoracic PHN who did not respond to various regimens including systemic and local medications as well as an intercostal nerve block, but showed an excellent result after treating with a single‐level T10 thoracic transforaminal epidural steroid injection.^34^ In 2018, Oh also reported a 82‐year‐old male patient with a treatment‐resistant thoracic PHN who was successfully treated using ultrasound‐guided pectoral II block.^35^ In a more recent research, Hu and coworkers treated 13 severe PHN patients who had VAS‐score of 9 or 10 with subcutaneous Botulinum Toxin‐A injection. The results suggested that this drug was an effective approach for reducing pain in patients with PHN. However, the authors did not mention whether prior to enrollment the patients were refractory to conventional treatments or not.^36^ In general, to date, the effective treatments for refractory PHN are still lacking, and seeking new methods are always necessary.

### 4.1 Complications

The subcutaneous injection of methylprednisolone in combination with lidocaine is an invasive intervention, thereby technique and medication‐associated complications can occur during or after procedure. Local complications such as abscesses, cutaneous atrophy, and scarring have been reported in previous investigations,^10^, ^30^ but they were not observed in our study. In our opinion, that was because we used only a small dose of methylprednisolone acetate (5‐10 mg) diluted in lidocaine (6‐12 mL), so these complications were minimized. In the present study, subcutaneous hemorrhage was commonly seen, and it was noted predominantly in older female patients. However, this bleeding was self‐limited and completely absorbed over time. Another frequent minor complication was pain at the injection points. We found that this pain only persisted for one to two initial intervention days, and all patients showed a good tolerance. Systemic complications: vertigo due to lidocaine intoxication, and weight gain caused by fluid retention effect of corticosteroid were also complained by a few patients, but they were minimal, transient, and rapidly reversible. Despite the appearance of the abovementioned adverse effects, they were generally mild and did not disrupt the course or success of the treatment.

### 4.2 Limitations

A major limitation in this research was the lack of a control group for comparison. However, as earlier described, prior to enrolling, all available conventional medications for PHN management had been prescribed for the patients, but they all showed no response or tolerance. Therefore, we did not establish control groups as patients were treated with the above drugs. Ideally, the standard control group in our study should have been treated with subcutaneous injection of saline solution alone (placebo), but it would have been impossible because of different reasons as mentioned in previous studies.^10^, ^11^, ^30^ An additional drawback was the small sample size. From May 2016 to October 2018, we screened a total of 43 refractory PHN patients, but they all agreed to participate in the research and none of them dropped out during the 12‐month follow‐up.

### 4.3 Advantages

Despite the abovementioned limitations, the method described in our study offers many advantages. Firstly, it is highly effective and safe. The excellent results reach 100% and exceed the initial expectations while the major complications are not seen, and all side effects are mild and reversible. Secondly, the intervention presents as a simple procedure because it only requires a syringe and a needle as an equipment that is easily available at any hospital or clinic. Additionally, any physician can implement this treatment method. Lastly, this treatment can be applied to the treatment of HZ at any site of the body. This point is superior to other interventions such as intrathecal or epidural injection; paravertebral block, which cannot be applied for PHN involving the trigeminal nerve.

**In conclusion**, the results indicate that subcutaneous injection of methylprednisolone acetate and lidocaine can be an effective and safe treatment for refractory PHN.

## CONFLICT OF INTEREST

The authors declare no potential conflict of interest.

## AUTHOR CONTRIBUTIONS

Conceptualization: Duc Thuan Nguyen, Thanh Chung Dang, and Quang An Nguyen

Data Curation: Duc Thuan Nguyen, Thanh Chung Dang, and Quang An Nguyen

Formal Analysis: Trung Duc Le, Thi Dung Hoang, Thi Ngoc Truong Tran, Ta Hai Ninh Duong, Van Tuan Nguyen, Van Quan Le, Tien Ung Hoang, Minh Tuan Duong, Dinh Son Nhu, and Viet Nga Phan

Investigation: Trung Duc Le, Thi Dung Hoang, Thi Ngoc Truong Tran, Ta Hai Ninh Duong, Van Tuan Nguyen, Van Quan Le, Tien Ung Hoang, Minh Tuan Duong, Dinh Son Nhu, and Viet Nga Phan

Methodology: Duc Thuan Nguyen, Thanh Chung Dang, and Quang An Nguyen

Resources: Trung Duc Le, Thi Dung Hoang, Thi Ngoc Truong Tran, Ta Hai Ninh Duong, Van Tuan Nguyen, Van Quan Le, Tien Ung Hoang, Minh Tuan Duong, Dinh Son Nhu, and Viet Nga Phan.

Supervision: Duc Thuan Nguyen, Thanh Chung Dang, and Quang An Nguyen

Writing—Original Draft Preparation: Duc Thuan Nguyen, Thanh Chung Dang, and Quang An Nguyen.

Writing—Review and Editing: Duc Thuan Nguyen, Thanh Chung Dang, and Quang An Nguyen

All authors have read and approved the final version of the manuscript.

Corresponding author, Duc Thuan Nguyen, had full access to all the data in this study and takes complete responsibility for the integrity of the data and the accuracy of the data analysis.

## TRANSPARENCY STATEMENT

Author Duc Thuan Nguyen (lead author) affirms that this manuscript is an honest, accurate, and transparent account of the study being reported; that no important aspects of the study have been omitted; and that any discrepancies from the study as planned have been explained.

## FINANCIAL STATEMENT

In our study, there are no funding sources or financial supports. Therefore, there is no conflict of interest.

## Supporting information


**FIGURE S1** The affected skin was divided into a chessboard for injection (Postherpetic Neuralgia in femoral area)

**FIGURE S2** Subcutaneous hemorrhage after injection (Postherpetic Neuralgia in cervical area)

**TABLE S1** Mean score of quality of life of study population before and after treatment

## Data Availability

The authors confirm that the data supporting the findings of this study are available within the article and its supplementary materials.

## References

[1] Cohen JI . Clinical practice: herpes zoster. N Engl J Med. 2013;369(3):255‐263. 10.1056/NEJMcp1302674. 23863052 PMC4789101

[2] Wood MJ . How should we measure pain in herpes zoster? Neurology. 1995;45(8):S61‐S62. 10.1212/WNL.45.12_Suppl_8.S61. 8545025

[3] Hadley GR , Gayle JA , Ripoll J , et al. Post‐herpetic neuralgia: a review. Curr Pain Headache Rep. 2016;20(3):17. 10.1007/s11916-016-0548-x. 26879875

[4] Lin CS , Lin YC , Lao HC , Chen CC . interventional treatments for postherpetic neuralgia: a systematic review. Pain Physician. 2019;22(3):209‐228. 31151330

[5] Serpell M , Gater A , Carroll S , Abetz‐Webb L , Mannan A , Johnson R . Burden of post‐herpetic neuralgia in a sample of UK residents aged 50 years or older: findings from the Zoster Quality of Life (ZQOL) study. Health Qual Life Outcomes. 2014;12:92. 10.1186/1477-7525-12-92. 24920439 PMC4063222

[6] Johnson RW , Bouhassira D , Kassianos G , Leplege A , Schmader KE , Weinke T . The impact of herpes zoster and post‐herpetic neuralgia on quality‐of‐life. BMC Med. 2010;8:37. 10.1186/1741-7015-8-37. 20565946 PMC2905321

[7] Mallick‐Searle T , Snodgrass B , Brant JM . Postherpetic neuralgia: epidemiology, pathophysiology, and pain management pharmacology. J Multidiscip Healthc. 2016;9:447‐454. 10.2147/JMDH.S106340. 27703368 PMC5036669

[8] Zorzoli E , Pica F , Masetti G , Franco E , Volpi A , Gabutti G . Herpes zoster in frail elderly patients: prevalence, impact, management, and preventive strategies. Aging Clin Exp Res. 2018;30(7):693‐702. 10.1007/s40520-018-0956-3. 29721782

[9] Arvin A . Aging, immunity, and the Varicella–Zoster Virus. N Engl J Med. 2005;352(22):2. 10.1056/NEJMp058091 15930416

[10] Epstein E . Triamcinolone‐procaine in the treatment of zoster and postzoster neuralgia. Calif Med. 1971;115(2):6‐10. 5563819 PMC1518011

[11] Ni J , Wang X , Tang Y , Yang L , Zeng Y , Guo Y . Subcutaneous injection of triamcinolone and lidocaine to prevent postherpetic neuralgia. Pain Physician. 2017;20(5):397‐403. 28727702

[12] Gan EY , Tian EA , Tey HL . Management of herpes zoster and post‐herpetic neuralgia. Am J Clin Dermatol. 2013;14(2):77‐85. 10.1007/s40257-013-0011-2. 23456596

[13] Heller GZ , Manuguerra M , Chow R . How to analyze the visual analogue scale: myths, truths and clinical relevance. Scand J Pain. 2016;13:67‐75. 10.1016/j.sjpain.2016.06.012. 28850536

[14] Ware Jr. JE , Sherbourne CD . The MOS 36‐item short‐form health survey (SF‐36). I. Conceptual framework and item selection. Med Care. 1992;30(6):473‐483. 1593914

[15] Chidiac C , Bruxelle J , Daures JP , et al. Characteristics of patients with herpes zoster on presentation to practitioners in France. Clin Infect Dis. 2001;33(1):62‐69. 10.1086/320884. 11389496

[16] Katz J , Cooper EM , Walther RR , Sweeney EW , Dworkin RH . Acute pain in herpes zoster and its impact on health‐related quality of life. Clin Infect Dis. 2004;39(3):342‐348. 10.1086/421942. 15307000

[17] Jeon YH . herpes zoster and postherpetic neuralgia: practical consideration for prevention and treatment. Korean J Pain. 2015;28(3):177‐184. 10.3344/kjp.2015.28.3.177. 26175877 PMC4500781

[18] Johnson RW , Rice AS . Clinical practice. Postherpetic neuralgia. N Engl J Med. 2014;371(16):1526‐1533. 10.1056/NEJMcp1403062. 25317872

[19] Attal N , Cruccu G , Baron R , et al. EFNS guidelines on the pharmacological treatment of neuropathic pain: 2010 revision. Eur J Neurol. 2010;17(9):1113‐e1188. 10.1111/j.1468-1331.2010.02999.x. 20402746

[20] Dubinsky RM , Kabbani H , El‐Chami Z , Boutwell C , Ali H , Quality Standards Subcommittee of the American Academy of, N . Practice parameter: treatment of postherpetic neuralgia: an evidence‐based report of the Quality Standards Subcommittee of the American Academy of Neurology. Neurology. 2004;63(6):959‐965. 10.1212/01.wnl.0000140708.62856.72. 15452284

[21] Horgas AL . Pain management in older adults. Nurs Clin North Am. 2017;52(4):e1‐e7. 10.1016/j.cnur.2017.08.001. 29080585

[22] Johnson RW , Wasner G , Saddier P , Baron R . Postherpetic neuralgia: epidemiology, pathophysiology and management. Expert Rev Neurother. 2007;7(11):1581‐1595. 10.1586/14737175.7.11.1581. 17997705

[23] Peng WW , Guo XL , Jin QQ , et al. Biological mechanism of post‐herpetic neuralgia: evidence from multiple patho‐psychophysiological measures. Eur J Pain. 2017;21(5):827‐842. 10.1002/ejp.985. 27977069

[24] Schlereth T , Heiland A , Breimhorst M , et al. Association between pain, central sensitization and anxiety in postherpetic neuralgia. Eur J Pain. 2015;19(2):193‐201. 10.1002/ejp.537. 25070366

[25] Golzari SE , Soleimanpour H , Mahmoodpoor A , Safari S , Ala A . Lidocaine and pain management in the emergency department: a review article. Anesth Pain Med. 2014;4(1):e15444. 10.5812/aapm.15444. 24660158 PMC3961016

[26] Sawynok J . Topical analgesics for neuropathic pain: preclinical exploration, clinical validation, future development. Eur J Pain. 2014;18(4):465‐481. 10.1002/j.1532-2149.2013.00400.x. 24108446

[27] Devor M , Govrin‐Lippmann R , Raber P . Corticosteroids suppress ectopic neural discharge originating in experimental neuromas. Pain. 1985;22(2):127‐137. 10.1016/0304-3959(85)90173-3. 4047699

[28] Theodoridis T , Krämer JW . Spinal Injection Techniques (Michelle James, Nettetal, & Germany, Trans.). Germany: GeorgThieme Verlag; 2007.

[29] Sehgal AD , Gardner WJ . Corticosteroids administered intradurally for relief of sciatica. Cleve Clin Q. 1960;27:198‐201. 10.3949/ccjm.27.4.198. 13749709

[30] Epstein E . Treatment of herpes zoster and postzoster neuralgia by subcutaneous injection of triamcinolone. Int J Dermatol. 1981;20(1):65‐68. 10.1111/j.1365-4362.1981.tb05299.x. 7203770

[31] Kikuchi A , Kotani N , Sato T , Takamura K , Sakai I , Matsuki A . Comparative therapeutic evaluation of intrathecal versus epidural methylprednisolone for long‐term analgesia in patients with intractable postherpetic neuralgia. Reg Anesth Pain Med. 1999;24(4):287‐293. 10.1016/s1098-7339(99)90101-3. 10445766

[32] Kotani N , Kushikata T , Hashimoto H , et al. Intrathecal methylprednisolone for intractable postherpetic neuralgia. N Engl J Med. 2000;343(21):1514‐1519. 10.1056/NEJM200011233432102. 11087880

[33] Rijsdijk M , van Wijck AJ , Meulenhoff PC , Kavelaars A , van der Tweel I , Kalkman CJ . No beneficial effect of intrathecal methylprednisolone acetate in postherpetic neuralgia patients. Eur J Pain. 2013;17(5):714‐723. 10.1002/j.1532-2149.2012.00233.x. 23059790

[34] Mehta P , Maher P , Singh JR . Treatment of postherpetic neuralgia using a thoracic transforaminal epidural steroid injection. PM R. 2015;7(4):443‐446. 10.1016/j.pmrj.2014.11.009. 25479280

[35] Oh DS . Pecs II block for intractable postherpetic neuralgia. J Anesth. 2018;32(3):460. 10.1007/s00540-018-2496-6. 29696382

[36] Hu Y , Zou L , Qi X , et al. Subcutaneous botulinum toxin—a injection for treating post‐herpetic neuralgia. Dermatol Ther. 2020;33:e13181. 10.1111/dth.13181. 31769900

