# Supplementary figures and images for: The effect of subcutaneous injection of methylprednisolone acetate and lidocaine for refractory postherpetic neuralgia: a prospective, observational study
Source: Health Sci Rep. 2021 Apr 8;4(2):e271. doi: 10.1002/hsr2.271 (PMC8031000; doi:10.1002/hsr2.271)

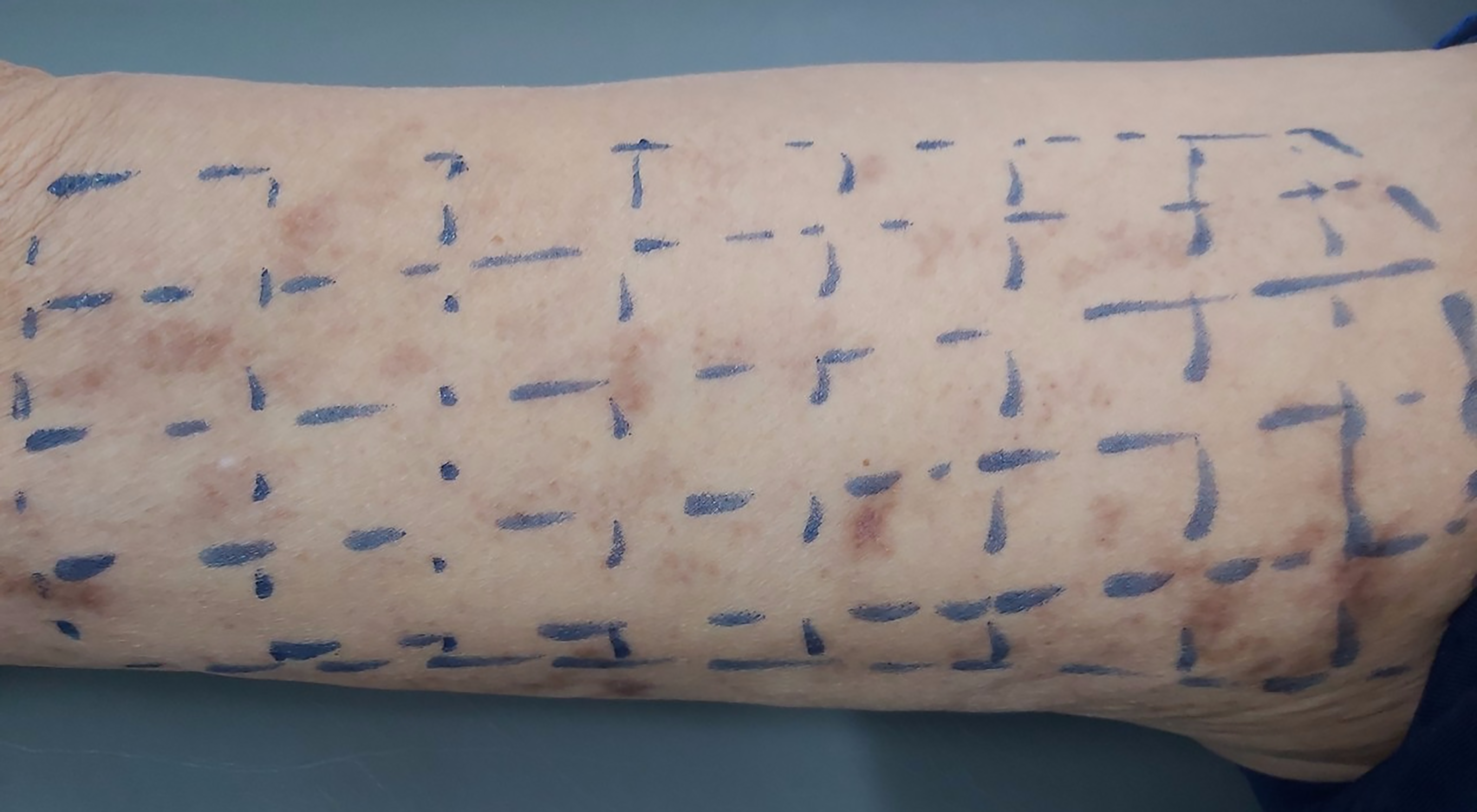

Supplement: Supplementary file 1 — FIGURE S1 The affected skin was divided into a chessboard for injection (Postherpetic Neuralgia in femoral area) [file HSR2-4-e271-s003.tif]

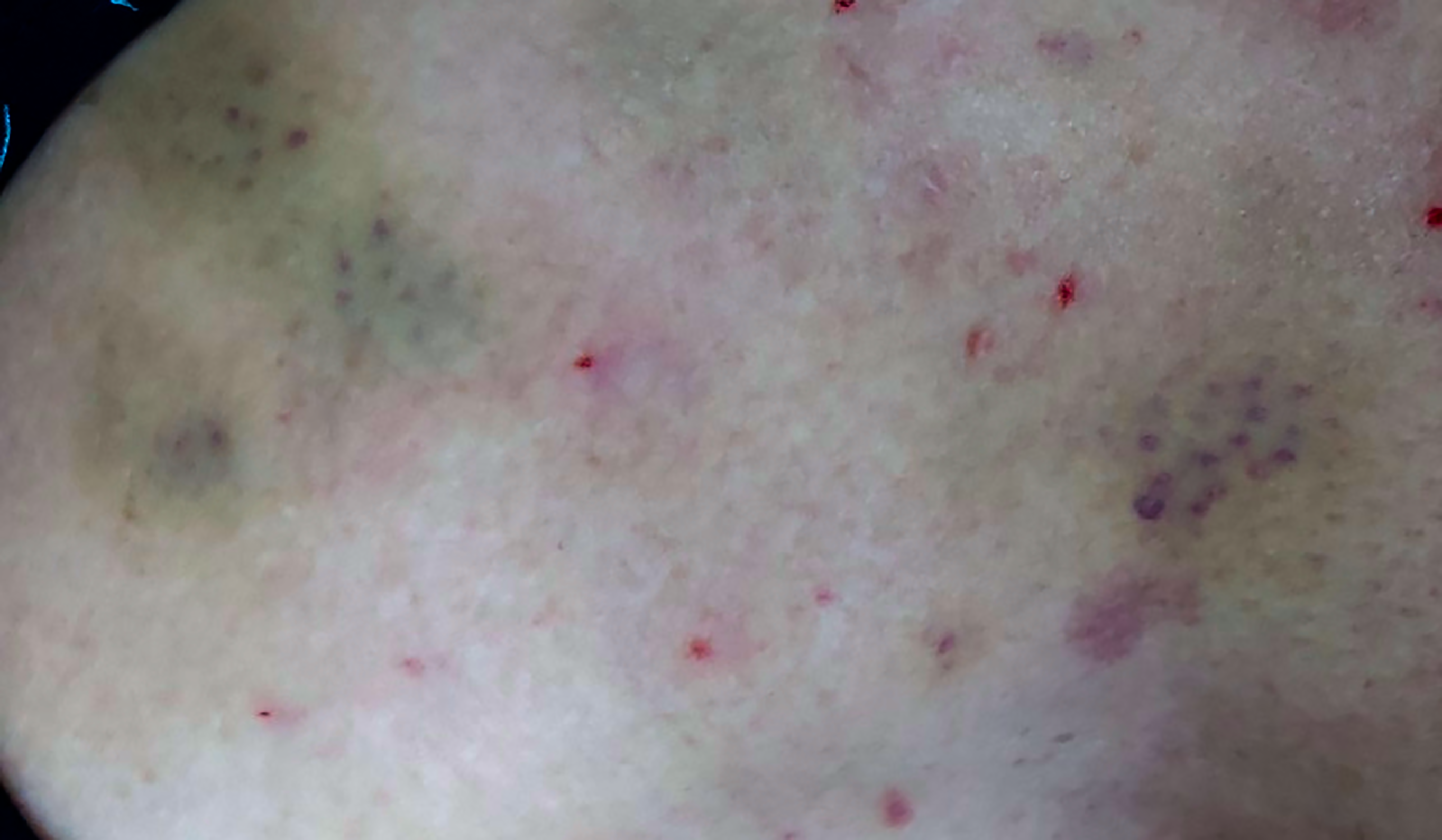

Supplement: Supplementary file 2 — FIGURE S2 Subcutaneous hemorrhage after injection (Postherpetic Neuralgia in cervical area) [file HSR2-4-e271-s002.tif]
