# Supplementary material for: The effect of subcutaneous injection of methylprednisolone acetate and lidocaine for refractory postherpetic neuralgia: a prospective, observational study
Source: Health Sci Rep. 2021 Apr 8;4(2):e271. doi: 10.1002/hsr2.271 (PMC8031000; doi:10.1002/hsr2.271)
Supplement: Supplementary file 3 — TABLE S1 Mean score of quality of life of study population before and after treatment [file HSR2-4-e271-s001.docx]

**Table S1. Mean score of quality of life of study population before and after treatment**

| Quality of life | Before treatment | 4 weeks after treatment | 6 months after treatment | 12 months after treatments | p-value* |
| --- | --- | --- | --- | --- | --- |
| Physical functioning (PF) | 21.27 ± 6.37 | 85.70 ± 5.83 | 86.74 ± 4.21 | 85.93 ± 5.90 | 0.0001 |
| Role emotional problems (REP) | 24.61 ± 13.19 | 88.57 ± 7.73 | 88.96 ± 6.24 | 88.38 ± 6.35 | 0.0001 |
| Role physical problems (RPP) | 22.38 ± 13.52 | 87.64 ± 6.75 | 87.50 ± 5.28 | 86.92 ± 5.42 | 0.0001 |
| Mental health (MH) | 19.07 ± 9.49 | 88.28 ± 7.11 | 89.72 ± 5.70 | 88.98 ± 6.05 | 0.0001 |
| Social functioning (SF) | 19.47 ± 12.29 | 87.79 ± 9.25 | 89.53 ± 6.06 | 88.37 ± 6.34 | 0.0001 |
| Bodily pain (BP) | 11.98 ± 9.15 | 87.97 ± 7.87 | 89.76 ± 6.02 | 89.01 ± 8.11 | 0.0001 |
| Vitality (VT) | 14.41 ± 7.65 | 87.55 ± 7.51 | 88.95 ± 4.16 | 87.44 ± 7.18 | 0.0001 |
| General health (GH) | 17.09 ± 7.23 | 86.63 ± 6.24 | 86.86 ± 3.94 | 85.81 ± 5.34 | 0.0001 |

* Wilcoxon signed rank sum test.
